# Cross cheek dumbbell-shaped radial forearm free flap for bilateral trismus release

**DOI:** 10.1080/23320885.2021.1977137

**Published:** 2021-09-18

**Authors:** Touqeer Hussain, Hasan Tahir, Obaid U. R. Rahman, Mirza Shehab Afzal Beg

**Affiliations:** a Department of Plastics and Reconstructive Surgery, Liaquat National Hospital, Karachi, Pakistan; b Department of Plastic and Reconstructive Surgery, Liaquat National Hospital and Medical College, Karachi, Pakistan

**Keywords:** Trismus, reconstruction, dumbbell-shaped forearm free flap

## Abstract

Trismus in post-radiotherapy patients is mostly secondary to fibrosis of buccal mucosa and muscles of mastication. After releasing trismus, mucosal defect needs to be covered with soft and supple tissue. We used a non-conventional method to cover this defect i.e. dumbbell-shape forearm free-flap based on a single radial artery.

## Introduction

The word ‘trismus’ is derived from a Greek word meaning prolonged and tetanic spasm of jaw muscles leading to restricted jaw opening [1]. The etiology of trismus is variable and is seen as a result of trauma, oral surgery, infection, or radiation in head and neck carcinomas [2]. This occurs might be due to oral sub-mucosal fibrosis (OSMF), fibrosis of the masseter muscle and/or, post-radiotherapy bone and cartilage necrosis (osteoradionecrosis). It may also be due to tumor infiltration of muscles of mastication or temporomandibular joint [3,4]. Due to this, patients develop difficulty in coping with routine activities including eating and drinking [4–7]. Trismus is graded by measuring inter-incisor distance (IID) according to the following criteria [8].

The prevalence of trismus is variably reported in the literature from 5% to 65% in head and neck cancers, with the latest data demonstrating 23.6% [4]. If these patients are identified beforehand and addressed, trismus can be prevented. Common non-surgical treatment modalities include jaw exercises, putting a gradually increasing number of tongue blades between anterior teeth and use of a dynamic bite opener have been used and reported efficacious [9]. Surgical release of trismus results in the variable size of the raw area on the oral sides of both cheeks needing coverage by flaps. Commonly used flaps to cover this raw area after trismus release includes two separate radial forearm free flap, double paddled RFFF on the same radial artery, tri-paddled ALTF with three separate sets of perforators, and single dumbbell-shaped RFFF [10–12]. Radial forearm fascio-cutaneous flaps are known for their pliability and good color match in head and neck reconstruction [13]. As described by Bhattacharya et al. [10], we aim to look into the effectiveness of a dumbbell shape RFFF by measuring inter-incisor distance.

Radial forearm flap is a dynamic flap with 9 to 17 septo-cutaneous perforators originating from the radial artery and traversing the lateral inter-muscular septum supplying the overlying skin with a maximum number supplying the distal forearm skin [10,14,15]. The flap can either be raised as a fascio-cutaneous or osteo-cutaneous flap [15].

## Material and methods

This is a retrospective study involving all patients who underwent bilateral cheek reconstruction with cross cheek dumbbell-shaped radial forearm free flap after bilateral trismus release over a period of 2 years (2017–2019). All cases were previously irradiated and tumor-free at the time of presentation. Inter-incisor distance was measured pre-operatively and graded. Physiotherapy of jaws and mouth opening exercise was started 2 weeks post-operatively. Inter incisor distance was measured at regular intervals up to 6 weeks post-operatively. Improvement in inter-incisor distance after surgery was used as a tool to measure the effectiveness of our procedure (Table 1) . Any complication of the procedure up to 6 weeks was observed noticed and addressed accordingly. The primary objectives of our study were to know the effectiveness of single cross cheek dumbbell-shaped RFFF in terms of flap survival, short & long-term complications, and donor site morbidity.

**Table 1.** Grades of trismus.

Grade of trismus	Inter-incisor distance
Grade 1	>35 mm
Grade 2	26–35 mm
Grade 3	16–25 mm
Grade 4	2–15 mm

### Surgical technique

**Figure 1.**
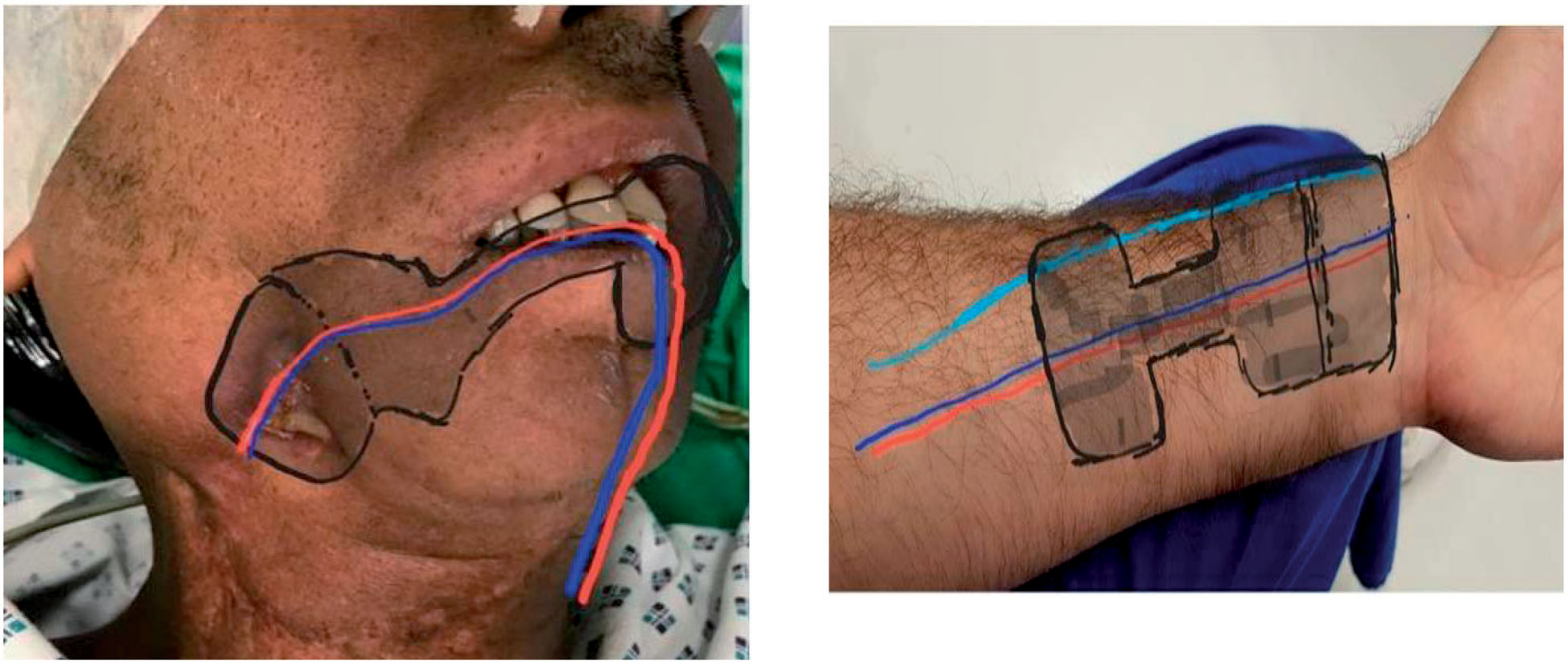
Schematic representation of flap design and harvest.

**Figure 2.**
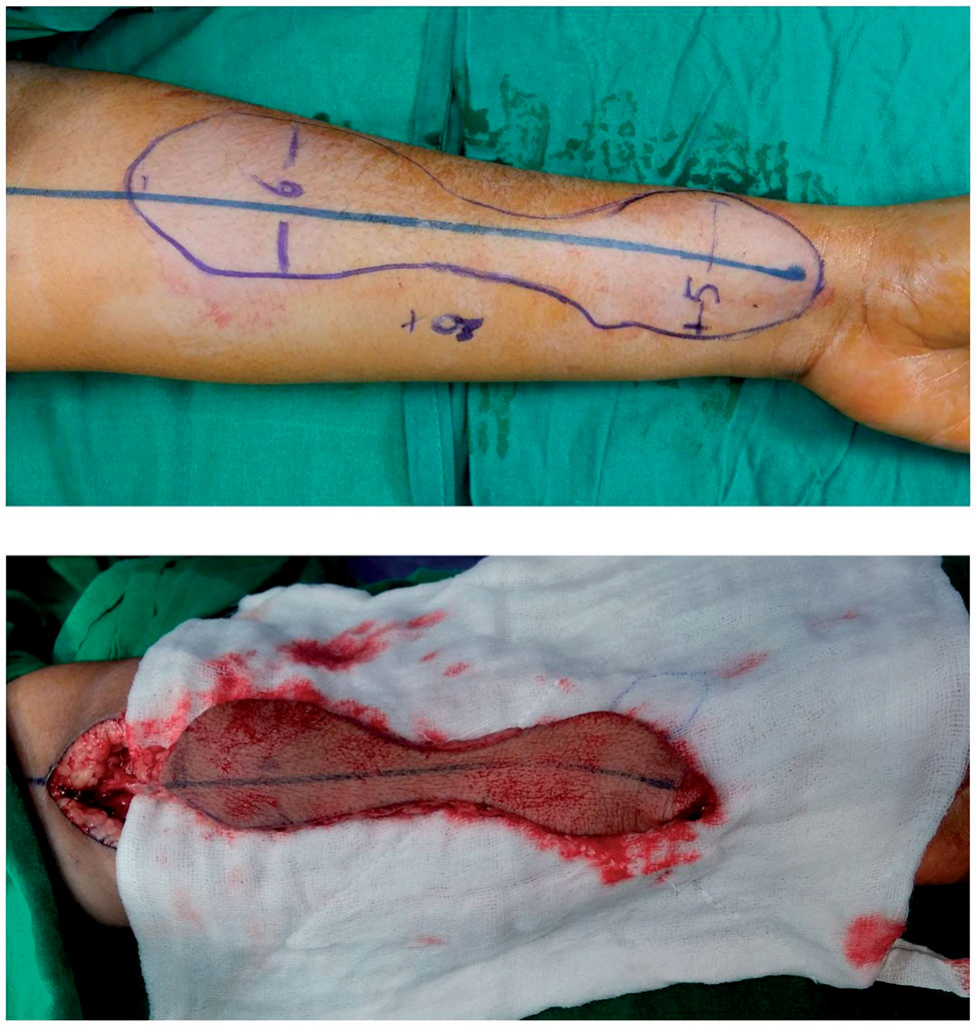
Flap design and harvest.

**Figure 3.**
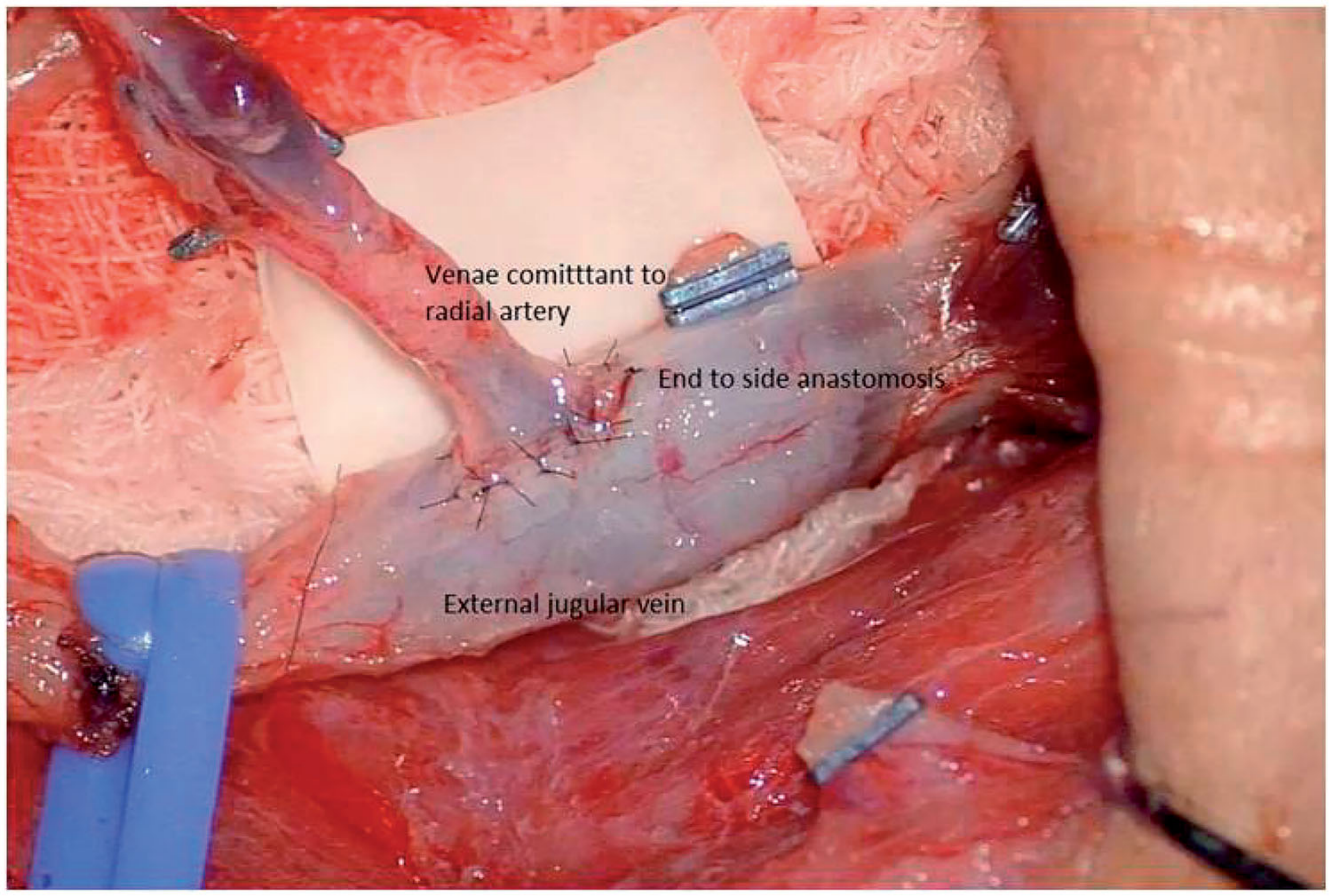
Micro vascular anastomosis.

**Figure 4.**
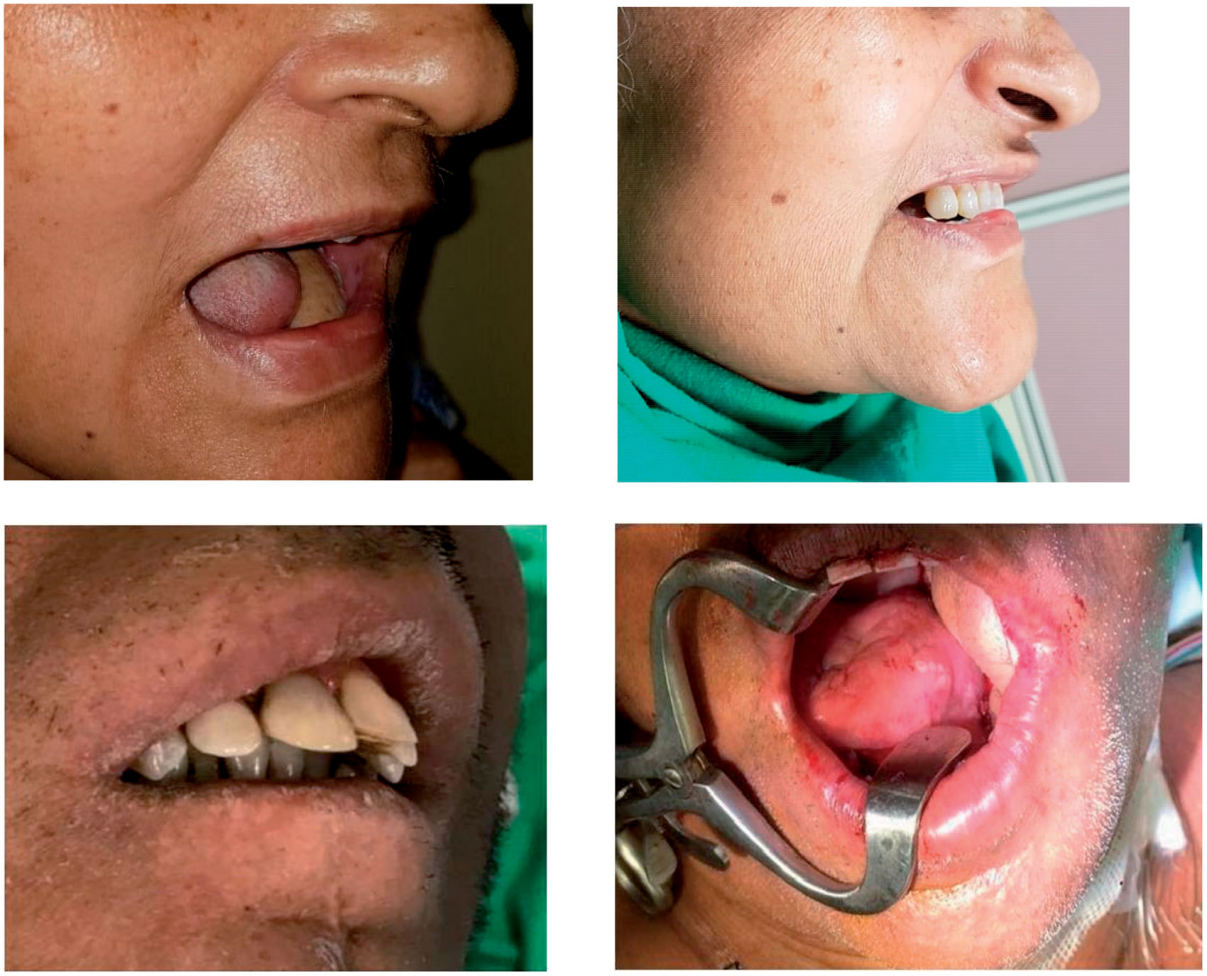
Pre and post-operative results.

Tumor-free cases with prior confirmation *via* PET scan were included in the study. In all the cases two teams participated simultaneously. The patient was placed in a supine position with the patient preferred forearm prepared for harvesting the flap (Figure 1) . Fibrotic tissue involving the gingivo-buccal mucosa was released, taking care not to damage the surrounding structures. Fibrotic buccinator, temporalis, and masseter muscles were released *via* myotomy. Per operative, measurements were carried out to make sure the adequate mouth opening to be beneficial for the patient in relieving the prime complaint. Intra-oral mucosal defect thus created at the end of trismus release was measured of both cheeks and a template was drawn and transposed on the forearm as per the intraoral defect (Figure 2). The RFFF was raised conventionally with the two dumbbells of the flap for the coverage of cheek defects created connected by a narrow skin paddle (Figure 3). Flap inset was done with absorbable sutures so that it covers the bilateral OSMF release sites and the mid-portion was incorporated in the gingivo-buccal sulcus and the pedicle was passed into the neck carefully by making a subcutaneous tunnel for micro-vascular anastomoses [Arterial anastomosis in the facial artery and vein into the internal jugular vein] (Figure 4). The donor site was covered with a skin graft.

## Results

A total of five patients was operated on between the period of 2017 and 2019. As per our experience, we found that there was a remarkable improvement in the IID when compared pre- and post-operatively, as explained in detail Table 2. The mean flap size was 16.2 ± 2.8 cm with 0.9 ± 0.4 cm central skin bridge. No donor site morbidity was observed. Both short and long-term outcomes were achieved as expected. None of our patients required secondary procedures. All patients were able to drink and can take a bolus. With physiotherapy, all continued to have desired IID and had no problems with speech.

**Table 2.** Comparison of pre and post-operative inter-incisor distance.

S. no.	Age	Gender	Addiction	Previous surgery	Pre-op IID	6 month post-operative IID	Complications (if any)
1.	37	Male	Nut chewer	Wide local excision of left buccal mucosa SCC + left MRND	1.4 cm	4.8 cm	SSI
2.	45	Male	Smoker	Wide local excision of right cheek SCC + Right MRND	0.9 cm	4.2 cm	None
3.	55	Male	Gutka chewer	Wide local excision of left buccal mucosa SCC tumor with marginal mandibulectomy	0.8 cm	4.3 cm	None
4.	40	Female	Nut chewer and smoker	Wide local excision of left buccal mucosa SCC and infra-structure maxillectomy	1.2 cm	4.5 cm	SSI
5.	52	Male	Smoker	Retromolartrigone wide local excision of SCC excision 3 years back	1.1 cm	4.2 cm	None

IID: inter-incisors distance; SCC: squamous cell carcinoma; MRND: modified radical neck dissection.

## Discussion

Trismus is known to complicate post-operative outcomes in oral cancer surgery. Most often the cause is found to be oral submucosal fibrosis and fibrosis of muscles of mastication. Wei et al. [16] used two separate RFFF for bilateral buccal mucosa reconstruction. This technique demands both upper limbs to be worked on for flap harvest, causes scars on both upper limbs, needs double vascular anastomosis (both flaps anastomosed separately) leading to longer operating time burdening both surgeon and patient, in contrast, our technicians use only a single forearm and single anastomosis. The utilization of two independent RFFF from the single radial artery was presented by Tsao et al. [17] splitting fascio-cutaneous flap into two, based on perforators. He marked distal flap in the standard fashion and the proximal skin paddle is designed in the middle third of the forearm, based on septo-cutaneous branches of the radial artery. He elevates the two flaps and subsequently divides them into two independent free flaps, like bilateral RFFF this demands two separate micro-vascular anastomoses. Jiang et al. [18] presented a tri-paddled anterolateral thigh flap with three independent sets of perforators to cover the trismus defect.

RFFF for bilateral cheek reconstruction, with skin paddle being accommodated in the lower gingivo labial sulcus has been described as causing the lower lip prolapse [17]. Bi-paddled RFFF from a single forearm was described in the journal of Oral Maxillofacial Surgery in 2007 by Lee JT et al. [19] skeletonized ‘bridge pedicle’ was placed under the anterior vestibule mucosa, predisposed to pedicle compression especially in cases with sub-mucous fibrosis while in our case a narrow skin paddle is used connecting both dumbbells for coverage of bridging pedicle providing supple tissue over connecting pedicle as well.

Bilateral tongue flaps have also been described previously to cover the trismus defect with significant morbidity including dysphasia, disarticulation, and aspiration [19].

In our study and other similar studies, it was found that there are many advantages of utilizing RFFF for reconstructing such defects. Dumbbell-shaped RFFF covers three anatomically distinct sites (Bilateral cheeks and gingivo-buccal sulcus) with a single flap and one microvascular anastomosis. Like every flap, dumbbell-shaped RFFF is not free of drawbacks. The first being the need for skin grafting for donor site wound coverage, needs microvascular expertise, risk of anastomotic failure, and need to prolong physiotherapy to avoid future recurrence of trismus.

## Conclusion

Single artery radial forearm free flap is more effective as compared to conventional methods. Our technique is time-efficient, has low cost, results in the adequate release of trismus, and needs single donor site utilization. Larger sample size with long-term follow-up on functional outcome is still required.
